# Pyrotinib versus pertuzumab with trastuzumab and taxane in HER2-positive metastatic breast cancer: a Chinese multicenter real-world study

**DOI:** 10.1093/oncolo/oyaf277

**Published:** 2025-09-09

**Authors:** Binliang Liu, Zongbi Yi, Can Tian, Jian Deng, Ronghua Feng, Zhe-Yu Hu, Ning Xie, Yongxin Li, Jiuda Zhao, Tao Wu, Quchang Ouyang

**Affiliations:** Department of Breast Cancer Medical Oncology, Hunan Cancer Hospital/The Affiliated Cancer Hospital of Xiangya School of Medicine, Central South University, Changsha 410013, China; Department of Radiation and Medical Oncology, Hubei Key Laboratory of Tumor Biological Behaviors, Hubei Cancer Clinical Study Center, Zhongnan Hospital of Wuhan University, Wuhan 430071, China; Department of Breast Cancer Medical Oncology, Hunan Cancer Hospital/The Affiliated Cancer Hospital of Xiangya School of Medicine, Central South University, Changsha 410013, China; Department of Thyroid Breast Surgery, The Second Affiliated Hospital, Hengyang Medical School, University of South China, Hengyang 421009, China; Department of Oncology, Changde Hospital, Xiangya School of Medicine, Central South University, Changde 415000, China; Department of Breast Cancer Medical Oncology, Hunan Cancer Hospital/The Affiliated Cancer Hospital of Xiangya School of Medicine, Central South University, Changsha 410013, China; Department of Breast Cancer Medical Oncology, Hunan Cancer Hospital/The Affiliated Cancer Hospital of Xiangya School of Medicine, Central South University, Changsha 410013, China; Department of Oncology, Hunan Institute of Schistosomiasis Control (The Third People’s Hospital of Hunan Province), Yueyang 414000, China; Breast Disease Diagnosis and Treatment Center, Affiliated Hospital of Qinghai University and Affiliated Cancer Hospital of Qinghai University, Xining 810000, China; Breast Disease Diagnosis and Treatment Center, Affiliated Hospital of Qinghai University and Affiliated Cancer Hospital of Qinghai University, Xining 810000, China; Department of Oncology, Changde Hospital, Xiangya School of Medicine, Central South University, Changde 415000, China; Department of Breast Cancer Medical Oncology, Hunan Cancer Hospital/The Affiliated Cancer Hospital of Xiangya School of Medicine, Central South University, Changsha 410013, China

**Keywords:** breast cancer, HER2-positive, first-line, THP, THPy, real-world study

## Abstract

**Importance:**

THP (trastuzumab + paclitaxel + pertuzumab) and THPy (trastuzumab + paclitaxel + pyrotinib) are widely used as first-line regimens for human epidermal growth factor receptor 2 (HER2)-positive metastatic breast cancer (MBC) in China. However, direct comparative data on their efficacy and safety remain scarce.

**Objective:**

To evaluate and compare the clinical outcomes of THPy and THP in the first-line treatment of HER2-positive MBC to guide clinical decision-making.

**Design:**

Real-world, multicenter retrospective study conducted from 2020 to 2024.

**Setting:**

Five large breast cancer centers in China.

**Participants:**

A total of 145 patients with HER2-positive MBC were included before propensity score matching (PSM). After PSM, 76 patients (38 in each group) were analyzed.

**Intervention(s) or Exposure(s):**

Patients received either THP (trastuzumab + paclitaxel + pertuzumab) or THPy (trastuzumab + paclitaxel + pyrotinib) as first-line treatment.

**Main Outcome(s) and Measure(s):**

The primary endpoint was progression-free survival (PFS). Secondary endpoints included objective response rate (ORR), disease control rate, and safety profile.

**Results:**

Before PSM, 145 patients were included, with the median follow-up of 9.4 months. The ORR was significantly higher in the THPy group (75.0%) compared to the THP group (56.8%; *P* = .023). Median PFS showed a trend favoring THPy (19.80 months vs 15.57 months; Log-rank *P* = .343). After PSM, 76 patients were matched, with the median PFS of 24.33 months versus 14.50 months for THPy and THP groups, respectively. Though the difference in PFS was not statistically significant (Log-rank *P* = .309), THPy demonstrated a higher ORR compared to THP (78.9% vs 57.9%; *P* = .048). Further subgroup analysis revealed greater benefits of THPy, particularly in patients with prior neoadjuvant therapy (hazard ratio [HR] = 0.261; 95% CI: 0.072-0.940). Regarding safety, THPy was associated with a higher incidence of grade 3/4 diarrhea (26.6% vs 3.1%) as well as increased rates of neutropenia, anemia, alanine aminotransferase elevation, fatigue, and peripheral neuropathy compared to THP.

**Conclusion and Relevance:**

Both THP and THPy are effective first-line options for HER2-positive MBC. While THPy demonstrates a higher ORR and a trend toward longer PFS, it also carries a higher incidence of adverse events, particularly diarrhea. These findings offer preliminary insights that may help inform treatment decisions, pending further validation in prospective studies.

Implications for Practice Both trastuzumab + pertuzumab + taxane (THP) and trastuzumab + pyrotinib + taxane (THPy) are recommended as first-line treatment options for HER2-positive metastatic breast cancer in Chinese guidelines. However, head-to-head comparisons between these regimens have been lacking. This multicenter real-world study shows that THPy yields a higher objective response rate and a trend toward longer progression-free survival, albeit with a higher incidence of gastrointestinal toxicity. These findings provide preliminary insights into optimizing frontline treatment selection and highlight the importance of individualized therapy decisions based on efficacy, safety, and patient characteristics. Prospective trials are warranted to validate these findings and further guide clinical practice.

## Introduction

Breast cancer remains one of the most prevalent malignancies among women worldwide, with approximately 15%-20% of cases classified as human epidermal growth factor receptor 2 (HER2)-positive.^1^ This molecular subtype is associated with more aggressive tumor biology, higher recurrence rates, and poorer prognosis.^2^ The advent of HER2-targeted therapies, including monoclonal antibodies and small-molecule tyrosine kinase inhibitors (TKIs), has significantly improved clinical outcomes for this population.^3^

In the first-line setting for HER2-positive advanced breast cancer, the CLEOPATRA trial^4^ established the combination of trastuzumab, pertuzumab, and docetaxel (THP) as the global standard of care. This multicenter, phase III study demonstrated that THP significantly prolonged both progression-free survival (PFS; 18.5 vs 12.4 months) and overall survival (OS; 57.1 vs 48.2 months) compared with trastuzumab and docetaxel (TH) alone.

Pyrotinib is an oral, irreversible pan-ErbB TKI that targets HER1, HER2, and HER4. Compared to reversible TKIs, it provides sustained inhibition of downstream signaling.^5^^,^^6^ Pyrotinib has shown robust efficacy and manageable safety in multiple phase II/III trials and is now approved in China for second-line treatment of HER2-positive metastatic breast cancer.^7^^,^^8^

Encouraging outcomes with TKI–trastuzumab combinations have been observed in trials, such as NeoALTTO,^9^ PHEDRA,^10^ and HER2CLIMB,^11^ especially in neoadjuvant or later-line contexts. Building on these findings, the phase III PHILA study^6^ evaluated pyrotinib plus trastuzumab and docetaxel (THPy) versus TH as first-line therapy for HER2-positive metastatic breast cancer. Conducted across 40 centers in China, the trial enrolled 590 untreated patients and reported a median PFS of 24.3 months in the THPy group versus 10.4 months in the TH group (HR = 0.41), with an Independent Review Committee confirming a PFS of 33.3 months. These results led to THPy being included as a recommended frontline regimen in Chinese clinical guidelines. However, the PHILA study was limited by the absence of OS data and a smaller sample size compared to CLEOPATRA (*n* = 808).^4^

Although both THP and THPy regimens have demonstrated efficacy, a direct head-to-head comparison is lacking. Moreover, differences in toxicity profiles—particularly the higher incidence of diarrhea with THPy—may influence patient adherence and quality of life. It remains unclear which regimen provides greater benefit in real-world clinical practice and for which subgroups.

To address these gaps, we conducted a multicenter retrospective study across 5 cancer centers in China to compare the efficacy and safety of THP versus THPy in the first-line treatment of HER2-positive metastatic breast cancer. Using propensity score matching (PSM) to minimize confounding, we provide real-world evidence to inform treatment decisions and support personalized therapeutic strategies.

## Methods

### Study design and patients

This was a retrospective, multicenter, observational study conducted across 5 leading breast cancer centers in China, in accordance with the Declaration of Helsinki, the International Conference on Harmonisation Good Clinical Practice (ICH-GCP) guidelines, and local ethical regulations. Ethical approval was obtained from the Ethics Committee and Institutional Review Board of Hunan Cancer Hospital (Approval No. SBQLL-2022-066). The study was registered at ClinicalTrials.gov (NCT05367739).

Eligible patients were those with HER2-positive advanced breast cancer who received either the THP regimen (trastuzumab + pertuzumab + paclitaxel/docetaxel) or the THPy regimen (trastuzumab + pyrotinib + paclitaxel/docetaxel) between June 2020 and May 2024. Inclusion criteria were age >18 years; histologically or cytologically confirmed locally recurrent or metastatic breast cancer; HER2-positive status (defined as immunohistochemistry [IHC] 3+ or fluorescence in situ hybridization positive); good general condition with an ECOG performance status of 0; and measurable disease according to RECIST v1.1 criteria.^12^ Patients scheduled to receive first-line therapy for advanced disease were included, defined as those who had not received prior systemic therapy for advanced disease or had disease recurrence more than 1 year after completion of adjuvant therapy. Patients with stable or asymptomatic brain metastases were also eligible.

All efficacy analyses were conducted based on a modified intention-to-treat population, which included patients who received at least one dose of the study treatment and had available follow-up or efficacy evaluation data. Patients were excluded if they had incomplete baseline or follow-up data or were lost to follow-up before any efficacy assessment. Patients who received any treatment other than THP or THPy regimens—such as trastuzumab monotherapy or antibody-drug conjugates (ADCs)—were also excluded.

### Endpoints

The primary endpoint was PFS, defined as the time from the first administration of medication to the first documented tumor progression or death from any cause. The secondary endpoints included the objective response rate (ORR), defined as the percentage of patients achieving a complete response (CR) or partial response (PR); disease control rate (DCR); defined as the percentage of patients achieving CR, PR, or stable disease (SD); and AEs. The date of onset of AEs and their severity were graded according to the National Cancer Institute Common Terminology Criteria for Adverse Events (CTCAE), version 5.0.

### Data collection

Patient demographic information and medical history were obtained from electronic medical records. Collected data included general information (age and sex), cancer-related details (disease stage, pathology, metastatic sites, and disease characteristics), treatment history (current and previous chemotherapy or targeted therapies, surgical and radiation therapy histories, and neoadjuvant therapy), and efficacy-related outcomes (treatment response, progression status, and date of the last follow-up). Visceral metastases were defined as the presence of metastases in any of the following organs: liver, lung, brain (including leptomeningeal involvement), bone marrow, or other vital organs that may pose life-threatening risks.

All participants underwent routine monitoring, including vital sign measurements, physical examinations, and laboratory tests. Efficacy and safety assessments were performed every 6 weeks, with imaging modalities (CT or MRI) selected according to the standard practices of each participating center. Importantly, this study did not interfere with clinical decision-making or delay any patient treatments.

### Propensity score matching

To minimize baseline imbalances between treatment groups, PSM was conducted. Propensity scores were derived using a logistic regression model, with baseline variables showing *P*-values < .2 serving as predictors. A 1:1 nearest-neighbor matching approach without replacement was used, with a caliper width of 0.25 standard deviations of the propensity score logit. The effectiveness of matching was evaluated through standardized mean difference plots and probability density visualizations before and after matching.

### Statistical analysis

Categorical variables were analyzed using Pearson’s χ^2^ test or Fisher’s exact test, as appropriate. PFS was assessed using Kaplan–Meier (K–M) survival analysis, with log-rank tests employed to compare survival curves. The Cox proportional hazards model was used to identify factors influencing PFS, presenting results as hazard ratios (HRs) with 95% CIs and corresponding *P*-values. Variables with *P*-values < .2 in the univariate analysis were included in the multivariate model to adjust for potential confounders.

All statistical tests were 2-sided, with *P*-values < .05 considered statistically significant. Analyses were performed using SPSS software (version 23, IBM SPSS Inc). K–M survival plots and risk tables were generated using the “survival” R package in R (version 3.6.0, Institute for Statistics and Mathematics). PSM and probability density analyses were conducted using R (version 4.3.0) in conjunction with the Storm Statistical Platform (www.medsta.cn/software).

## Result

### Baseline characteristics before PSM

**Figure 1.**
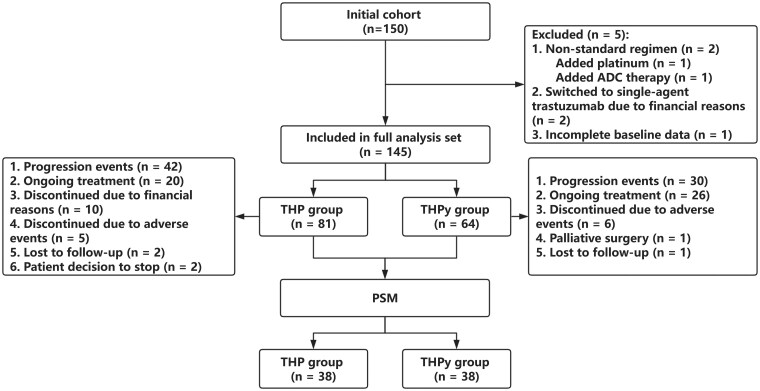
Flow diagram of patient enrollment, exclusion, treatment status, and propensity score matching (PSM). A total of 150 HER2-positive metastatic breast cancer patients were initially screened. Five patients were excluded due to non-standard regimens (*n* = 2), early treatment switch to single-agent trastuzumab for financial reasons (*n* = 2), or incomplete baseline data (*n* = 1). A total of 145 patients were included in the full analysis set, with 81 receiving THP and 64 receiving THPy. Detailed treatment outcomes for each group are shown. After PSM, 76 patients (38 in each group) were included in the final matched analysis.

A total of 150 patients with HER2-positive metastatic breast cancer were initially screened for eligibility. Five patients were excluded from the analysis due to non-standard regimens (*n* = 2, including one case each with added platinum and ADC therapy), early treatment switch to single-agent trastuzumab due to financial reasons (*n* = 2), and incomplete baseline data (*n* = 1). As a result, 145 patients were included in the full analysis set, comprising 81 patients in the THP group and 64 patients in the THPy group. A detailed flow diagram of patient selection and treatment status is presented in Figure 1.

Baseline characteristics before and after PSM were presented in Table 1. The median age of the study population before PSM was 53 years (range: 27-85), with a similar age distribution in the THP (27-85) and THPy (29-73) groups. At diagnosis, 40% of patients had de novo stage IV disease, while the remaining 60% had recurrent metastatic disease after treatment for early-stage breast cancer. Invasive ductal carcinoma accounted for 93.8% of all cases, and 51.7% were hormone receptor-negative (estrogen receptor-negative [ER-] and progesterone receptor-­negative [PgR-]).

**Table 1.** Demographics and clinicopathologic characteristics.

	Before PSM							After PSM						
	Total		THP		THPy		*P*	Total		THP		THPy		*P*
	(*N* = 145)		(*N* = 81)		(*N* = 64)			(*N* = 76)		(*N* = 38)		(*N* = 38)		
	*N*	%	*N*	%	*N*	%		*N*	%	*N*	%	*N*	%	
**Age**							.219							.093
** ≤50**	51	35.2	32	39.5	19	29.7		27	35.5	17	44.7	10	26.3	
** >50**	94	64.8	49	60.5	45	70.3		49	64.5	21	55.3	28	73.7	
**Side**							.115							.634
** Left**	82	56.6	41	50.6	41	64.1		48	63.2	25	65.8	23	60.5	
** Right**	60	41.4	37	45.7	23	35.9		28	36.8	13	34.2	15	39.5	
** Bilateral**	3	2.1	3	3.7	0	0.0		0	0.0	0	0.0	0	0.0	
**Disease characteristic**							.009							.353
** Recurrent disease**	87	60.0	41	50.6	46	71.9		44	57.9	20	52.6	24	63.2	
** De novo stage IV**	58	40.0	40	49.4	18	28.1		32	42.1	18	47.4	14	36.8	
**Pathology**							.290^a^							1.000^a^
** Invasive ductal carcinoma**	136	93.8	78	96.3	58	90.6		71	93.4	36	94.7	35	92.1	
** Non-invasive ductal carcinoma**	9	6.2	3	3.7	6	9.4		5	6.6	2	5.3	3	7.9	
**Stage at initial diagnosis**							.033							.669
** I**	3	2.1	1	1.2	2	3.1		2	2.6	1	2.6	1	2.6	
** II**	40	27.6	16	19.8	24	37.5		17	22.4	9	23.7	8	21.1	
** III**	44	30.3	24	29.6	20	31.3		25	32.9	10	26.3	15	39.5	
** IV**	58	40.0	40	49.4	18	28.1		32	42.1	18	47.4	14	36.8	
**Pathological immunohistochemistry**							.111							.946
** ER+/PgR+**	51	35.2	34	42.0	17	26.6		25	32.9	13	34.2	12	31.6	
** ER+/PgR-**	13	9.0	9	11.1	4	6.3		4	5.3	2	5.3	2	5.3	
** ER-/PgR+**	6	4.1	3	3.7	3	4.7		3	3.9	1	2.6	2	5.3	
** ER-/PgR-**	75	51.7	35	43.2	40	62.5		44	57.9	22	57.9	22	57.9	
**Neoadjuvant therapy**							.379							.629
** None^b^**	94	64.8	50	61.7	44	68.8		50	65.8	24	63.2	26	68.4	
** Yes**	51	35.2	31	38.3	20	31.3		26	34.2	14	36.8	12	31.6	
**Neoadjuvant/adjuvant targeted therapy regimen**							.354							.952
** None**	82	56.6	42	51.9	40	62.5		45	59.2	22	57.9	23	60.5	
** Single-target**	23	15.9	13	16.0	10	15.6		13	17.1	7	18.4	6	15.8	
** Double-target**	40	27.6	26	32.1	14	21.9		18	23.7	9	23.7	9	23.7	
**Postoperative radiotherapy**							.542							.464
** None**	99	68.3	57	70.4	42	65.6		51	67.1	27	71.1	24	63.2	
** Yes**	46	31.7	24	29.6	22	34.4		25	32.9	11	28.9	14	36.8	
**Disease-free survival**							.373							.356
** <2 years**	71	49.0	37	45.7	34	53.1		42	55.3	19	50.0	23	60.5	
** >2 years**	74	51.0	44	54.3	30	46.9		34	44.7	19	50.0	15	39.5	
**Metastatic sites**														
** Visceral**	87	60.0	44	54.3	43	67.2	.116	50	65.8	27	71.1	23	60.5	.333
** Chest**	31	21.4	16	19.8	15	23.4	.591	15	19.7	8	21.1	7	18.4	.773
** Liver**	43	29.7	32	39.5	11	17.2	.003	24	31.6	14	36.8	10	26.3	.324
** Lung**	52	35.9	29	35.8	23	35.9	.987	33	43.4	19	50.0	14	36.8	.247
** Bone**	57	39.3	35	43.2	22	34.4	.279	31	40.8	16	42.1	15	39.5	.815
** Brain**	25	17.2	9	11.1	16	25.0	.028	12	15.8	6	15.8	6	15.8	1.000^a^
**The type of taxane used**							.027							.491
** Docetaxel**	60	41.4	27	33.3	33	51.6		39	51.3	18	47.4	21	55.3	
** Paclitaxel**	85	58.6	54	66.7	31	48.4		37	48.7	20	52.6	17	44.8	

Abbreviations: ER, estrogen receptor; PgR, progesterone receptor.

a Continuity correction.

b Including de novo stage IV patients.

Regarding prior treatments, 35.2% had received neoadjuvant therapy, 15.9% had undergone trastuzumab monotherapy as part of neoadjuvant or adjuvant therapy, and 27.6% had received dual HER2-targeted neoadjuvant/adjuvant therapy with trastuzumab and pertuzumab. Visceral metastases were present in 60% of patients, with brain metastases occurring in 17.2%.

Patients newly diagnosed with advanced breast cancer were more likely to receive the THP regimen, as reflected by a significantly higher proportion in the THP group compared to the THPy group (49.4% vs 28.1%, *P* = .009). Conversely, patients with brain metastases were more likely to receive the THPy regimen, evidenced by the higher proportion in the THPy group compared to the THP group (25.0% vs 11.1%, *P* = .028).

### Efficacy analysis before PSM

Among the THP group, 42 patients experienced disease progression, 20 remained on treatment at the time of data cutoff, while treatment was discontinued in 10 due to financial reasons, 5 due to adverse events (AEs), 2 were lost to follow-up, and 2 discontinued treatment by personal decision. In the THPy group, 30 patients progressed, 26 were still receiving treatment, 6 discontinued due to AEs, 1 underwent palliative surgery, and 1 was lost to follow-up (Figure 1).

**Figure 2.**
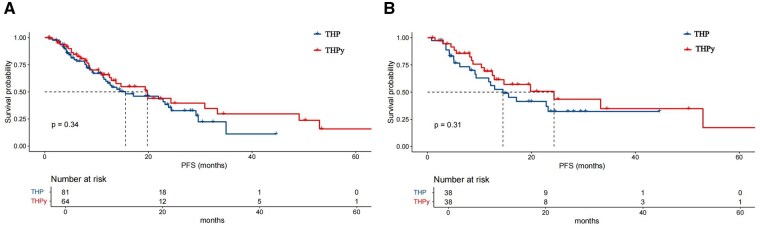
Kaplan–Meier progression-free survival (PFS) curves comparing the THP and THPy treatment groups. (A) PFS analysis for the overall cohort. (B) PFS analysis for the propensity score–matched (PSM) cohort. The number at risk at different time points is shown below each graph. Log-rank test P-values indicate no significant difference in PFS between the two groups (P = .34 in A, P = .31 in B).

**Figure 3.**
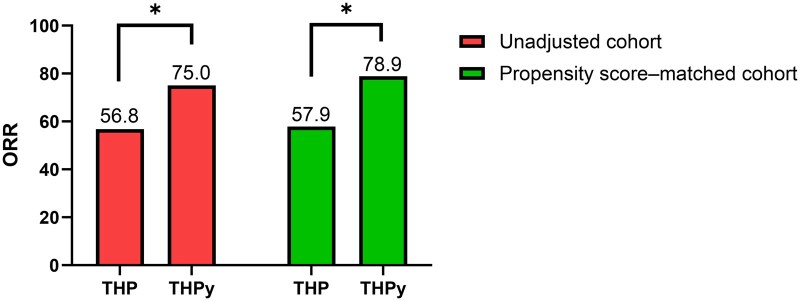
Objective response rate (ORR) comparison between the THP and THPy treatment groups. The left two bars represent the crude cohort, while the right two bars represent the propensity score–matched cohort. ORR is significantly higher in the THPy group compared to the THP group in both the crude cohort (75.0% vs 56.8%) and the propensity score–matched cohort (78.9% vs 57.9%), with statistical significance indicated by the asterisks (*).

With a median follow-up time of 9.4 months, the median PFS before PSM was 19.80 months (95% CI: 12.71-26.89) in the THPy group, compared to 15.57 months (95% CI: 6.13-25.01) in the THP group, indicating a trend favoring THPy (Log-rank *P* = .343, Figure 2A). The ORR was significantly higher in the THPy group (75.0%) than in the THP group (*P* = .023, Figure 3). The DCR was 93.8% in both groups. In the THP group, CR was achieved by 4.9% of patients, PR by 51.9%, SD by 37.0%, and PD by 6.2%. In the THPy group, CR was observed in 7.8% of patients, PR in 67.2%, SD in 18.8%, and PD in 6.3%. Detailed efficacy outcomes before PSM are summarized in Table 2.

**Table 2.** Evaluation of efficacy.

Variable	Before PSM			After PSM		
	THP (*N* = 81)	THPy (*N* = 64)	*P*-value	THP (*N* = 38)	THPy (*N* = 38)	*P*-value
**Median PFS**	15.57 (6.13-25.01)	19.80 (12.71-26.89)	.343	14.50 (8.40-20.60)	24.33 (9.03-39.63)	.309
**Events—No. (%)**	42 (51.9)	30 (46.9)	—	20 (52.6)	17 (44.7)	—
**Best overall response—No. (%)**			.110			.251
** Complete response**	4 (4.9)	5 (7.8)	—	3 (6.7)	5 (13.2)	—
** Partial response**	42 (51.9)	43 (67.2)	—	19 (50.0)	25 (65.8)	—
** Stable disease**	30 (37.0)	12 (18.8)	—	13 (34.2)	6 (15.8)	—
** Progressive disease**	5 (6.2)	4 (6.3)	—	3 (7.9)	2 (5.3)	—
**ORR—No. (%)**	46 (56.8)	48 (75.0)	.023	22 (57.9)	30 (78.9)	.048
**DCR—No. (%)**	76 (93.8)	60 (93.8)	1.000^a^	35 (92.1)	36 (94.7)	1.000^a^

Abbreviations: DCR, disease control rate; ORR, objective response rate; PFS, progression-free survival.

a Continuity correction.

### Predictive factors for efficacy

Univariate Cox regression analysis identified the absence of prior neoadjuvant therapy as a significant risk factor for poorer outcomes in the THP group (HR = 2.132, 95% CI: 1.142-3.981, *P* = .017). This finding remained significant in multivariate analysis (HR = 2.445, 95% CI: 1.126-5.310, *P* = .024). No significant predictive factors were identified in the THPy group (Table S1).

### AEs before PSM

AEs observed before PSM are summarized in Table 3. In the THP group, the most common AEs were leukopenia (59.3%), anemia (42.0%), neutropenia (30.9%), nausea/appetite loss (27.2%), and thrombocytopenia (24.7%). Grade 3/4 AEs included leukopenia (17.2%), neutropenia (17.3%), anemia (2.5%), thrombocytopenia (1.2%), and hyponatremia (2.5%).

**Table 3.** Adverse events of all grades before PSM.

AEs	THP (*N* = 81)						THPy (*N* = 64)					
	All grades		Grade 3 (*n*, %)		Grade 4 (*n*, %)		All grades		Grade 3 (*n*, %)		Grade 4 (*n*, %)	
	No. of patients	%	No. of patients	%	No. of patients	%	No. of patients	%	No. of patients	%	No. of patients	%
**Hematologic**												
** Leukopenia**	48	59.3	10	12.3	4	4.9	39	60.9	14	21.9	3	4.7
** Neutropenia**	25	30.9	9	11.1	5	6.2	34	53.1	8	12.5	9	14.1
** Anemia**	34	42.0	2	2.5	0	0.0	41	64.1	3	4.7	2	3.1
** Thrombocytopenia**	20	24.7	1	1.2	0	0.0	8	12.5	1	1.6	1	1.6
** lymphopenia**	10	12.3	1	1.2	0	0.0	17	26.6	4	6.3	1	1.6
**Nonhematologic**												
** AST increased**	7	8.6	0	0.0	0	0.0	7	10.9	1	1.6	0	0.0
** ALT increased**	10	12.3	1	1.2	0	0.0	20	31.3	4	6.3	0	0.0
** Hypercholesterolemia**	10	12.3	0	0.0	0	0.0	11	17.2	0	0.0	0	0.0
** Hypertriglyceridemia**	9	11.1	0	0.0	0	0.0	11	17.2	0	0.0	0	0.0
** Diarrhea**	2	2.5	0	0.0	0	0.0	46	71.9	17	26.6	2	3.1
** Nausea/decreased appetite**	22	27.2	0	0.0	0	0.0	18	28.1	0	0.0	0	0.0
** Pain**	7	8.6	0	0.0	0	0.0	15	23.4	1	1.6	0	0.0
** Peripheral neuropathy**	9	11.1	0	0.0	0	0.0	17	26.6	1	1.6	0	0.0
** Creatinine elevation**	10	12.3	0	0.0	0	0.0	12	18.8	0	0.0	0	0.0
** Uric acid elevation**	19	23.5	0	0.0	0	0.0	12	18.8	0	0.0	0	0.0
** Hyperglycemia**	6	7.4	0	0.0	0	0.0	6	9.4	0	0.0	0	0.0
** Hypocalcemia**	5	6.2	0	0.0	0	0.0	16	25.0	0	0.0	0	0.0
** Hypokalemia**	14	17.3	0	0.0	0	0.0	18	28.1	1	1.6	0	0.0
** Hyponatremia**	11	13.6	2	2.5	0	0.0	8	12.5	0	0.0	0	0.0
** Blood bilirubin increased**	1	1.2	0	0.0	0	0.0	4	6.3	0	0.0	0	0.0
** Fatigue**	11	13.6	0	0.0	0	0.0	24	37.5	1	1.6	0	0.0
** Pyrexia**	6	7.4	0	0.0	0	0.0	2	3.1	1	1.6	0	0.0
** Infection**	4	4.9	0	0.0	0	0.0	1	1.6	0	0.0	0	0.0
** Dizziness**	5	6.2	0	0.0	0	0.0	2	3.1	0	0.0	0	0.0
** Vomiting**	1	1.2	0	0.0	0	0.0	16	25.0	4	6.3	0	0.0
** Hypoalbuminemia**	14	17.3	0	0.0	0	0.0	27	42.2	1	1.6	0	0.0
** Myocardial enzyme elevation**	5	6.2	0	0.0	0	0.0	2	3.1	0	0.0	0	0.0
** Myocardial ischemia**	3	3.7	0	0.0	0	0.0	0	0.0	0	0.0	0	0.0
** Arrhythmia**	9	11.1	0	0.0	0	0.0	5	7.8	0	0.0	0	0.0
** Constipation**	7	8.6	0	0.0	0	0.0	3	4.7	0	0.0	0	0.0
** Low spirits**	7	8.6	0	0.0	0	0.0	13	20.3	0	0.0	0	0.0

Abbreviations: AEs, adverse events; ALT, alanine aminotransferase; AST, aspartate transaminase.

In the THPy group, the most frequent AEs were diarrhea (71.9%), anemia (64.1%), leukopenia (60.9%), neutropenia (53.1%), and hypoalbuminemia (42.2%). Grade 3/4 AEs included diarrhea (29.7%), leukopenia (26.6%), neutropenia (26.6%), anemia (7.8%), alanine aminotransferase (ALT) elevation (6.3%), and vomiting (6.3%).

When comparing the THP and THPy regimens, notable differences were observed in hematological and non-hematological toxicities. For hematological toxicity, the incidence of leukopenia in the THP group was 59.3%, with 17.2% of cases being grade 3 or higher. In the THPy group, leukopenia occurred in 60.9% of patients, with a higher proportion (26.6%) at grade 3 or above. Neutropenia was more prevalent in the THPy group (53.1% vs 30.9%), with a significantly higher grade 4 incidence (14.1% vs 6.2%). Additionally, anemia was more frequent in the THPy group (64.1% vs 42.0%). For non-hematological toxicity, diarrhea was significantly more common in the THPy group, affecting 71.9% of patients compared to 2.5% in the THP group. Grade 3 or higher diarrhea occurred in 26.6% of the THPy group, versus 3.1% in the THP group. Elevated ALT levels were also more frequent in the THPy group (31.3% vs 12.3%), with grade 3 ALT elevation observed in 6.3% of patients compared to 1.2% in the THP group. Fatigue was reported by 37.5% of patients in the THPy group, compared to 13.6% in the THP group. Regarding neurotoxicity, peripheral neuropathy was more common in the THPy group (26.6% vs 11.1%). Hypokalemia was also reported more frequently in the THPy group (28.1% vs 17.3%).

These findings suggest that while the overall incidence of AEs was comparable between the THP and THPy regimens, the THPy regimen was associated with a higher incidence and greater severity of specific AEs, particularly diarrhea, ALT elevation, and fatigue.

### Baseline characteristics after PSM

Propensity scores were derived using baseline variables with *P*-values < .2 as predictors (see Table S2 for the list of variables). Following PSM, a total of 76 patients were included after PSM, with 38 in each group. There were no significant differences in the characteristics between the 2 groups (*P* > .05) (Table 1). The probability density analysis plot is presented in Figure S1 (see online supplementary material for a color version of this figure). The median age was 53 years (range: 31-73). Of the patients, 42.1% were diagnosed with advanced-stage disease, 57.9% were hormone receptor-negative (ER- and PR-), and 34.2% had previously undergone neoadjuvant therapy. Additionally, 17.1% had received single-target neoadjuvant/adjuvant trastuzumab therapy, while 23.7% had received dual-target neoadjuvant/adjuvant therapy with both trastuzumab and pertuzumab. Furthermore, 65.8% of patients had visceral metastases, including 15.8% with brain metastases.

### Efficacy analysis after PSM

With the median follow-up time of 11.3 months, the median PFS was notably longer in the THPy group at 24.33 months (95% CI: 9.03-39.63), compared to 14.50 months (95% CI: 8.40-20.60) in the THP group (Log-rank *P* = .309, Figure 2B), indicating a trend toward better outcomes in the THPy group. Regarding treatment efficacy, the ORR was significantly higher in the THPy group at 78.9%, compared to 57.9% in the THP group (*P* = .048, Figure 3). The DCR was consistently high in both groups, with 92.1% in the THP group and 94.7% in the THPy group. The distribution of treatment responses demonstrated that in the THP group, CR was achieved in 6.7% of patients, PR in 50.0%, SD in 34.2%, and PD in 7.9%. In contrast, the THPy group showed higher rates of CR (13.2%), and PR (65.8%), with SD observed in 15.8%, and PD in 5.3% of patients (Table 2).

**Figure 4.**
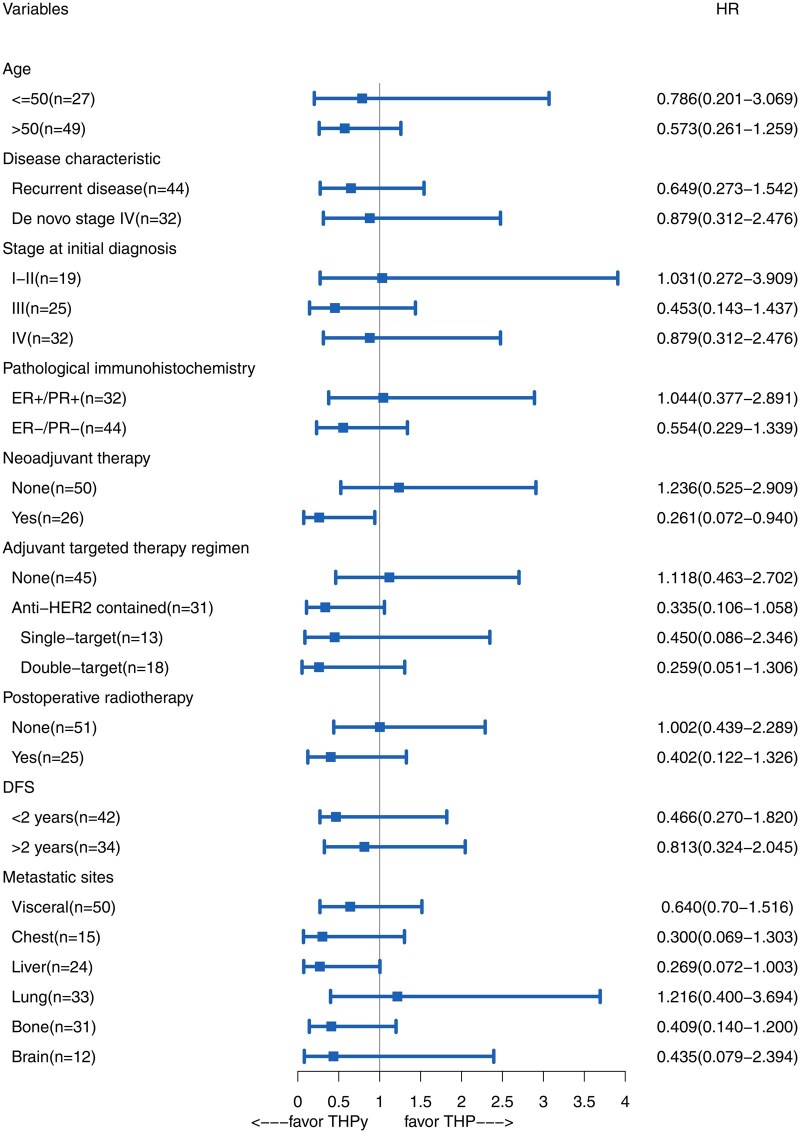
Forest plot displaying the hazard ratios (HRs) and 95% confidence intervals (CIs) for various clinical and pathological factors in relation to survival outcomes. Significant associations are observed in patients who received neoadjuvant therapy (HR = 0.261, 95% CI: 0.072-0.940).

In the subgroup analysis, the majority of subgroups showed a trend of greater benefit from THPy. However, only patients who had previously received neoadjuvant therapy demonstrated a significantly improved efficacy with THPy compared to the THP regimen (HR = 0.261, 95% CI: 0.072-0.940). The forest plot depicting the relationship between treatment regimens and efficacy in each subgroup is shown in Figure 4.

## Discussion

The CLEOPATRA study^4^ established trastuzumab, pertuzumab, and docetaxel (THP) as the global standard first-line treatment for HER2-positive MBC, demonstrating significant improvements in both PFS and OS. The subsequent PERUSE study^13^ further supported this strategy using paclitaxel, reinforcing the role of taxane-based dual HER2-targeted regimens as first-line options.

Pyrotinib is an oral, irreversible pan-HER TKI developed in China. It has demonstrated favorable efficacy and safety across multiple phase II and III trials,^7^^,^^8^ establishing itself as a standard second-line therapy in HER2-positive MBC. The combination of pyrotinib and trastuzumab offers several mechanistic advantages: (1) Dual blockade: Trastuzumab binds to the extracellular domain IV of HER2, while pyrotinib targets the intracellular tyrosine kinase domain, enhancing inhibition of HER2 signaling pathways.^14–16^ (2) HER2 downregulation: Pyrotinib promotes HER2 ubiquitination and membrane degradation without impairing trastuzumab-mediated antibody-dependent cell-mediated cytotoxicity.^15^^,^^17^ and (3) Blood–brain barrier penetration: TKIs can cross the blood–brain barrier, potentially improving outcomes in patients with brain metastases.^18^

The ongoing debate surrounding the choice of frontline treatment regimens for HER2-positive MBC underscores the need for further clinical data validation. This study represents the largest real-world comparison of THP and THPy as first-line treatments for HER2-positive MBC in China. Its primary significance lies in exploring the differences in efficacy and safety between these 2 regimens in clinical practice, as well as identifying patient subgroups most likely to benefit from each treatment. Although cross-trial comparisons should be interpreted with caution due to differences in study populations and settings, the PHILA study^6^ reported a median PFS of 24.3 months with the THPy regimen, which showed a trend toward longer PFS compared to the 18.7 months reported in CLEOPATRA.^4^ Similarly, the ORRs in PHILA and CLEOPATRA were 83% and 80.25%, respectively. These findings highlight the need for prospective, head-to-head studies to further evaluate the potential benefits of combining small- and large-molecule HER2-targeted agents.

In earlier studies comparing HP plus chemotherapy versus HPy plus chemotherapy, a small-sample Chinese multicenter trial that included only 24 patients reported a trend toward longer PFS with HP plus chemotherapy (22.90 months vs 14.46 months; *P* = .057) in the frontline setting for HER2-positive advanced breast cancer.^19^ A meta-analysis encompassing 22 studies^20^ and a network meta-analysis incorporating 20 randomized controlled trials^21^ affirmed that the pyrotinib + trastuzumab + docetaxel regimen showed the longest PFS among first-line treatments for HER2-positive advanced MBC, achieving the highest ORR. However, in terms of OS, trastuzumab combined with pertuzumab may yield better outcomes.^20^ Further Bayesian network meta-analysis corroborated that the pyrotinib regimen ranked highest in PFS and ORR, providing the best efficacy and safety balance.^21^

However, it is important to note that in clinical practice, the choice of treatment regimen may be influenced by factors, such as patient preference, financial considerations, and the presence of brain metastases, potentially leading to selection bias between groups. This highlights the necessity of using PSM in our study to enhance the reliability of the conclusions. After matching, our results indicated that in the treatment of HER2-positive advanced breast cancer, the THPy regimen achieved a significantly higher ORR compared to the THP regimen (THPy: 78.9% vs THP: 57.9%). The THPy regimen also demonstrated a numerical advantage in PFS (24.33 months vs 14.50 months), although the difference was not statistically significant. Both regimens are recommended in Chinese clinical guidelines, underscoring their comparable relevance as first-line treatment options for HER2-positive metastatic breast cancer.

Brain metastases occur in approximately 30%-50% of patients with HER2-positive MBC^22^ posing a major treatment challenge. Previous studies suggest that TKI drugs not only treat brain metastases effectively but may also prevent their occurrence.^23^ The HER2CLIMB study^11^^,^^24^ demonstrated that among patients with brain metastases, the addition of tucatinib to trastuzumab and capecitabine extended OS by 9.1 months (21.6 months; 95% CI, 18.1-28.5 vs 12.5 months; 95% CI, 11.2-16.9) with a median follow-up of 29.6 months. Significant benefits were also observed in central nervous system PFS (CNS-PFS), intracranial ORR, intracranial duration of response, and reduced risk of developing new brain metastases. In the prospective phase II PERMEATE study,^25^ 78 patients enrolled from 8 centers explored the efficacy of pyrotinib combined with capecitabine in treating newly diagnosed or recurrent brain metastases in MBC patients. Among the 78 patients, 8 achieved complete remission of intracranial lesions. In patients without prior local radiotherapy, the ORR was 74.6%, with a median PFS of 10.9 months and a median OS of 35.9 months, demonstrating pyrotinib’s efficacy for brain metastases. Real-world studies have also demonstrated promising intracranial activity of HER2-TKIs in patients with brain metastases.^26^

Currently, ADCs such as such as trastuzumab deruxtecan (T-DXd), have demonstrated promising efficacy in patients with brain metastases.^27–29^ Novel treatment strategies involving combinations of ADCs, TKIs, and monoclonal antibodies are currently under investigation and may reshape the future therapeutic landscape for these patients.

In terms of adverse reactions, multiple studies have found that the THPy regimen has a significantly higher incidence of gastrointestinal AEs, particularly diarrhea, compared to the THP regimen. However, hematological and other toxicities are similar between the 2 regimens.^19^^,^^20^ Diarrhea is a notable side effect associated with all HER2-TKIs, primarily due to EGFR inhibition, which is widely expressed in the gastrointestinal mucosa.^30^ Diarrhea, particularly during the first 1-2 months, is a significant tolerability issue for HER2-TKIs.^14^ Retrospective studies have reported that 34.3% of patients in the chemotherapy + HPy group experienced grade 3/4 diarrhea, which is substantially higher than the 3.0% observed in the chemotherapy + HP group (*P* < .001). These findings align with the grade 3/4 diarrhea rates of 26.6% and 3.1% in our THPy and THP groups, respectively. Furthermore, our study also demonstrated that THPy has a higher incidence of other AEs, including neutropenia, anemia, elevated ALT, fatigue, and peripheral neuropathy, compared to THP. These findings underscore the need for careful monitoring and management of adverse reactions when using the THPy regimen.

Several limitations of this study should be acknowledged. First, as a retrospective real-world analysis, the findings cannot be directly compared to those of prospective randomized controlled trials, which offer higher levels of evidence. Second, although PSM was employed to adjust for baseline imbalances, the post-matching sample size was relatively small (*n* = 76), which may have introduced selection bias and limited the statistical power of the analysis. Third, the median follow-up duration in this study was shorter than the observed median PFS, indicating that progression events had not yet fully matured and potentially affecting the robustness of the survival estimates. Moreover, differences in baseline disease burden between groups—such as a higher proportion of advanced-stage patients in the THP group and more brain metastases in the THPy group—may have introduced residual confounding despite PSM adjustment. In addition, as an exploratory real-world study, our findings require validation through larger prospective clinical trials. Lastly, patient-reported outcomes and quality-of-life (QoL) data were not collected, limiting our ability to comprehensively evaluate the tolerability and patient experience associated with each regimen.

Taken together, both THP and THPy regimens demonstrated clinical efficacy in the first-line treatment of HER2-positive metastatic breast cancer, with THPy showing a trend toward improved PFS and a significantly higher ORR. However, this potential benefit comes at the cost of increased toxicity, most notably grade 3/4 diarrhea, hematologic adverse events, and liver enzyme elevation. While these toxicities were generally manageable, their higher incidence with THPy may negatively impact patients’ QoL, especially in real-world settings. Moreover, the cost and accessibility of targeted agents, particularly pyrotinib, may vary across regions and healthcare systems. In the absence of direct pharmacoeconomic data, clinicians must weigh the trade-offs between efficacy and tolerability, taking into account patient comorbidities, prior treatments, preferences, and socioeconomic context.

Future prospective studies incorporating QoL measurements and cost-effectiveness analyses are warranted to better inform individualized treatment decisions between monoclonal antibody-based and TKI-based dual HER2-targeted regimens.

## Conclusion

Both the THP and THPy regimens demonstrated clinical activity in the first-line treatment of HER2-positive metastatic breast cancer. The THPy regimen was associated with a significantly higher objective response rate and a trend toward improved PFS compared to THP, although accompanied by higher rates of treatment-related adverse events, particularly diarrhea and hematologic toxicities. Given the retrospective design, relatively short follow-up, and immature PFS data, these results should be interpreted with caution. Rather than supporting immediate changes in clinical practice, these findings provide a basis for future prospective, randomized studies to more definitively assess the comparative efficacy, safety, quality of life, and cost-effectiveness of these 2 regimens.

## Supplementary Material

oyaf277_Supplementary_Data

## Data Availability

All data that support the findings of this study are accessible from the corresponding author upon reasonable request.
