# Supplementary material for: Pyrotinib versus pertuzumab with trastuzumab and taxane in HER2-positive metastatic breast cancer: a Chinese multicenter real-world study
Source: Oncologist. 2025 Sep 9;30(10):oyaf277. doi: 10.1093/oncolo/oyaf277 (PMC12497480; doi:10.1093/oncolo/oyaf277)
Supplement: oyaf277_Supplementary_Data [file oyaf277_supplementary_data.zip › Supplementary Table 1 and 2.docx]

Supplementary Table 1

| Variable | THP (N=81) | | | |  | THPy (N=64) | | | |
| --- | --- | --- | --- | --- | --- | --- | --- | --- | --- |
|  | Univariate Cox model | | Multivariate Cox model | |  | Univariate Cox model | | Multivariate Cox model | |
|  | HR (95% CI) | p-value | HR (95% CI) | p-value |  | HR (95% CI) | p-value | HR (95% CI) | p-value |
| Age |  |  |  |  |  |  |  |  |  |
| ≤50 | 1.260 (0.673-2.357) | 0.47 |  |  |  | 1.003 (0.444-2.264) | 0.995 |  |  |
| ＞50 | Ref | Ref |  |  |  | Ref | Ref |  |  |
| Side |  |  |  |  |  |  |  |  |  |
| Left | 0.545 (0.126-2.369) | 0.418 |  |  |  | 0.958 (0.448-2.049) | 0.912 |  |  |
| Right | 0.397 (0.121-2.309) | 0.529 |  |  |  | Ref | Ref |  |  |
| Bilateral | Ref | Ref |  |  |  | / | / |  |  |
| Disease characteristic |  |  |  |  |  | / | / |  |  |
| Recurrent disease | 1.206 (0.653-2.230) | 0.549 |  |  |  | 0.885 (0.391-2.003) | 0.769 |  |  |
| De novo stage IV | Ref | Ref |  |  |  | Ref | Ref |  |  |
| Pathology |  |  |  |  |  |  |  |  |  |
| Invasive ductal carcinoma | 1.895 (0.581-6.183) | **0.289** | 0.618 (0.171-2.229) | 0.462 |  | 1.662 (0.493-5.607) | 0.413 |  |  |
| Non-Invasive ductal carcinoma | Ref | Ref | Ref | Ref |  | Ref | Ref |  |  |
| Stage at initial diagnosis |  | 0.835 |  |  |  |  | 0.808 |  |  |
| I-II | 0.822 (0.373-1.811) | 0.626 |  |  |  | 1.238 (0.521-2.943) | 0.628 |  |  |
| III | 0.834 (0.407-1.711) | 0.621 |  |  |  | 0.952 (0.343-2.643) | 0.924 |  |  |
| IV | Ref | Ref |  |  |  | Ref | Ref |  |  |
| Pathological immunohistochemistry |  | 0.731 |  |  |  |  | 0.359 |  | 0.327 |
| ER+/PR+ | 0.802 (0.416-1.547) | 0.511 |  |  |  | 1.575 (0.713-3.479) | **0.261** | 2.426 (0.785-7.500) | 0.124 |
| ER+/PR- | 0.591 (0.198-1.764) | 0.345 |  |  |  | 0.565 (0.127-2.519) | 0.454 | 0.676 (0.145-3.145) | 0.619 |
| ER-/PR+ | 1.437 (0.185-11.167) | 0.729 |  |  |  | 0.365 (0.047-2.835) | 0.335 | 0.564 (0.068-4.668) | 0.596 |
| ER-/PR- | Ref | Ref |  |  |  | Ref | Ref | Ref | Ref |
| Neoadjuvant therapy (None vs Yes) | 2.132 (1.142-3.981) | **0.017** | 2.445 (1.126-5.310) | 0.024 |  | 0.808 (0.345-1.890) | 0.623 |  |  |
| Neoadjuvant/Adjuvant targeted therapy regimen |  | 0.425 |  | 0.89 |  |  | **0.203** |  | 0.552 |
| None | Ref | Ref |  |  |  | Ref | Ref | Ref | Ref |
| Single-target | 1.114 (0.489-2.535) | 0.797 | 0.811 (0.340-1.939) | 0.638 |  | 2.010 (0.838-4.824) | **0.118** | 1.081 (0.330-3.544) | 0.897 |
| Double-target | 1.601 (0.785-3.265) | **0.195** | 0.875 (0.359-2.130) | 0.768 |  | 0.720 (0.209-2.474) | 0.601 | 0.515 (0.146-1.818) | 0.303 |
| Postoperative radiotherapy (None vs Yes) | 0.999 (0.522-1.910) | 0.996 |  |  |  | 0.925 (0.420-2.038) | 0.847 |  |  |
| DFS |  |  |  |  |  |  |  |  |  |
| <2 years | 0.933 (0.502-1.733) | 0.825 |  |  |  | 1.384 (0.670-2.859) | 0.379 |  |  |
| >2 years | Ref | Ref |  |  |  | Ref | Ref |  |  |
| Metastatic sites |  |  |  |  |  |  |  |  |  |
| Visceral | 0.982 (0.534-1.805) | 0.953 |  |  |  | 1.134 (0.515-2.494) | 0.755 |  |  |
| Chest | 1.457 (0.726-2.926) | **0.290** | 1.830 (0.870-3.848) | 0.111 |  | 1.370 (0.638-2.942) | 0.420 |  |  |
| Liver | 1.220 (0.654-2.276) | 0.533 |  |  |  | 0.524 (0.182-1.509) | **0.231** | 0.477 (0.146-1.818) | 0.19 |
| Lung | 0.757 (0.391-1.465) | 0.757 |  |  |  | 1.191 (0.566-2.510) | 0.645 |  |  |
| Bone | 1.258 (0.690-2.325) | 0.465 |  |  |  | 0.498 (0.213-1.164) | **0.108** | 0.500 (0.189-1.318) | 0.161 |
| Brain | 1.620 (0.630-4.164) | 0.317 |  |  |  | 1.783 (0.758-4.192) | **0.185** | 1,928 (0.784-4.740) | 0.153 |
| Variables with p<0.3p<0.3 in the univariate Cox analysis were included in the multivariate Cox analysis. | | | | | | | | | |

Supplementary Table 2

| **Variable** | **Type** | Included in PSM |
| --- | --- | --- |
| Side | Categorical | Yes |
| Disease characteristic | Categorical | Yes |
| Stage at initial diagnosis | Categorical | Yes |
| Pathological immunohistochemistry | Categorical | Yes |
| Metastatic sites |  |  |
| Visceral |  |  |
| Liver | Binary | Yes |
| Brain | Binary | Yes |
| The type of taxane used | Binary | Yes |
