# Supplementary figures and images for: Pyrotinib versus pertuzumab with trastuzumab and taxane in HER2-positive metastatic breast cancer: a Chinese multicenter real-world study
Source: Oncologist. 2025 Sep 9;30(10):oyaf277. doi: 10.1093/oncolo/oyaf277 (PMC12497480; doi:10.1093/oncolo/oyaf277)

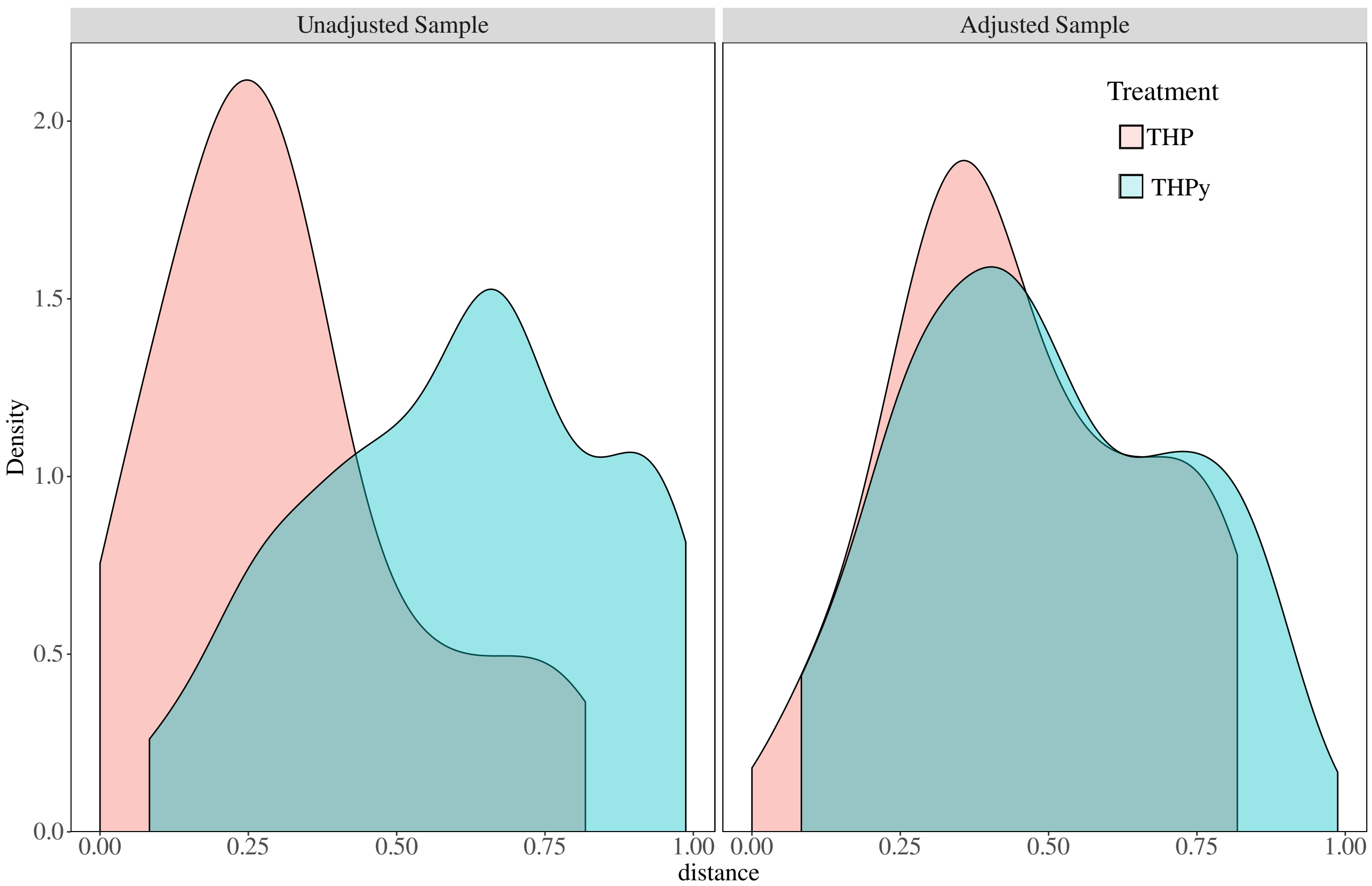

Supplement: oyaf277_Supplementary_Data [file oyaf277_supplementary_data.zip › Supplementary Figure 1.pdf]
